# Characterizing the respiratory-induced mechanical stimulation at the maxillary sinus floor following sinus augmentation by computational fluid dynamics

**DOI:** 10.3389/fbioe.2022.885130

**Published:** 2022-07-26

**Authors:** Qing Li, Zhongyu Wang, Chao Wang, Hom-Lay Wang

**Affiliations:** ^1^ Center of Digital Dentistry, Second Clinical Division, Peking University School and Hospital of Stomatology and National Center of Stomatology and National Clinical Research Center for Oral Diseases and National Engineering Research Center of Oral Biomaterials and Digital Medical Devices, Beijing, China; ^2^ Key Laboratory of Biomechanics and Mechanobiology, Ministry of Education, Beijing Advanced Innovation Center for Biomedical Engineering, School of Biological Science and Medical Engineering, School of Engineering Medicine, Beihang University, Beijing, China; ^3^ Department of Periodontics and Oral Medicine, University of Michigan School of Dentistry, Ann Arbor, MI, United States

**Keywords:** respiratory airflow, maxillary sinus floor augmentation, re-pneumatization, biomechanics, fluid dynamics

## Abstract

**Background:** The relationship between maxillary sinus pneumatization and respiratory-induced fluid mechanics remains unclear. The purpose of this study was to simulate and measure the respiratory-induced mechanical stimulation at the sinus floor under different respiratory conditions and to investigate its potential effect on the elevated sinus following sinus-lifting procedures.

**Methods:** The nasal airway together with the bilateral maxillary sinuses of the selected patient was segmented and digitally modeled from a computed tomographic image. The sinus floors of the models were elevated by simulated sinus augmentations using computer-aided design. The numerical simulations of sinus fluid motion under different respiratory conditions were performed using a computational fluid dynamics (CFD) algorithm. Sinus wall shear stress and static pressure on the pre-surgical and altered sinus floors were examined and quantitatively compared.

**Results:** Streamlines with minimum airflow velocity were visualized in the sinus. The sinus floor pressure and the wall shear stress increased with the elevated inlet flow rate, but the magnitude of these mechanical stimulations remained at a negligible level. The surgical technique and elevated height had no significant influence on the wall pressure and the fluid mechanics.

**Conclusion:** This study shows that respiratory-induced mechanical stimulation in the sinus floor is negligible before and after sinus augmentation.

## Introduction

Insufficient bone volume is a common problem encountered in the rehabilitation of the edentulous posterior maxilla with implant-supported prostheses. Bone volume is limited following tooth extraction due to maxillary sinus pneumatization together with alveolar bone resorption (Sharan and Madjar, 2006, 2008; Van der Weijden et al., 2009; Covani et al., 2011; Tan et al., 2012; Wagner et al., 2017). Maxillary sinus augmentation, either lateral window or transcrestal approach, is a well-documented approach to increase vertical height in the sinus cavity (Zhou et al., 2021). Nonetheless, a significant volumetric reduction of the placed graft was often observed over time (Berberi et al., 2015; Gultekin et al., 2016). However, the mechanism of graft resorption remains unclear (Tepper et al., 2002; Butaric et al., 2010).

Multiple factors have been associated with increased sinus volume, these include but are not limited to: surgical method, compression force during graft filling, initial ridge height and width, mechanical stimulation of airflow, and bone surface area in direct contact with graft material (Wallace and Froum, 2003; Kirmeier et al., 2008; Shanbhag et al., 2014). From a fluid biomechanical perspective, load-induced intraosseous pressure and fluid shearing stress are considered important mechanical cues to promote bone marrow stem cell osteogenesis and bone repair (Wittkowske et al., 2016; Stavenschi et al., 2018; Yong et al., 2020). Breathing, sneezing, sniffing, other motion induced airflow, and pressure drop along the nasal airspace will exert mechanical stimulation to the grafting area in the sinus. Although respiratory airflow is generally accepted as a mechanical factor affecting the outcome of bone grafting (Kurbel et al., 2004), there is a lack of understanding if these respiratory-induced mechanical stimulations (e.g., wall pressure and wall shear stress) will cause sinus cavity expansion or graft resorption.

Respiratory physiology and pathology are strongly dependent on the airflow inside the nasal cavity. The technical difficulty of quantitative analysis of the sinus cavity is due to the complicated airway passages and significant individual differences in nasal anatomy. As a result, the usual diagnostic analysis tools are often ineffective. Fortunately, with the rapid development of computer technology, computational fluid dynamics (CFD) has the capacity to link airflow characteristics in the human nasal airway to the actual effects of maxillary sinus lift. Furthermore, the computed nasal geometry can be virtually modified to reflect the predicted results of the proposed surgical technique (Chung et al., 2016).

Hence, in this study, the nasal airway together with maxillary sinuses was segmented and digitally modeled. The CFD model was used to numerically simulate the airflow movement under different respiratory conditions. By calculating and quantitatively comparing the wall shear stress and static pressure of the sinus floor before and after the change, the mechanical stimulation exerted by the respiratory process on the sinus floor was predicted. Therefore, the purpose of this study was to simulate and measure the respiratory-induced mechanical stimulation at the sinus floor under different respiratory conditions and to investigate its potential effect on the elevated sinus following sinus-lifting procedures.

## Materials and methods

### Digital modeling

The study was approved by the ethical committee of the School and Hospital of Stomatology, Peking University (PKUSSIRB-201838121). The digital models of the nasal cavity and maxillary sinuses were obtained from a 30-year-old systemically healthy male patient with a missing maxillary left first molar and with inadequate bone height at the edentulous region. No sinus inflammation and cyst were found. The patient has given informed consent to the acquisition, modification, and analysis of his radiological data. The cone beam computed tomography (CBCT) data files (scanning parameters are as follows: spatial resolution: 512 × 512 pixels, pixel width: 0.098 mm, cross-sectional slices: 653, slice thickness: 0.398 mm, DICOM format images were obtained) of the craniomaxillofacial tissue were loaded to the medical imaging software, Mimics 18.0 (Materialise, Leuven, Belgium), and the image of the nasal airspace and the maxillary sinuses were extracted and segmented to build an airspace model. Then the reconstructed STL model was imported to Geomagic Studio 2013 (Geomagic, North Carolina, United States) for surface extraction and solid model conversion. Sinus augmentation was simulated by computer-aided design software (Solidworks Premium 2020; United States).

**FIGURE 1:**
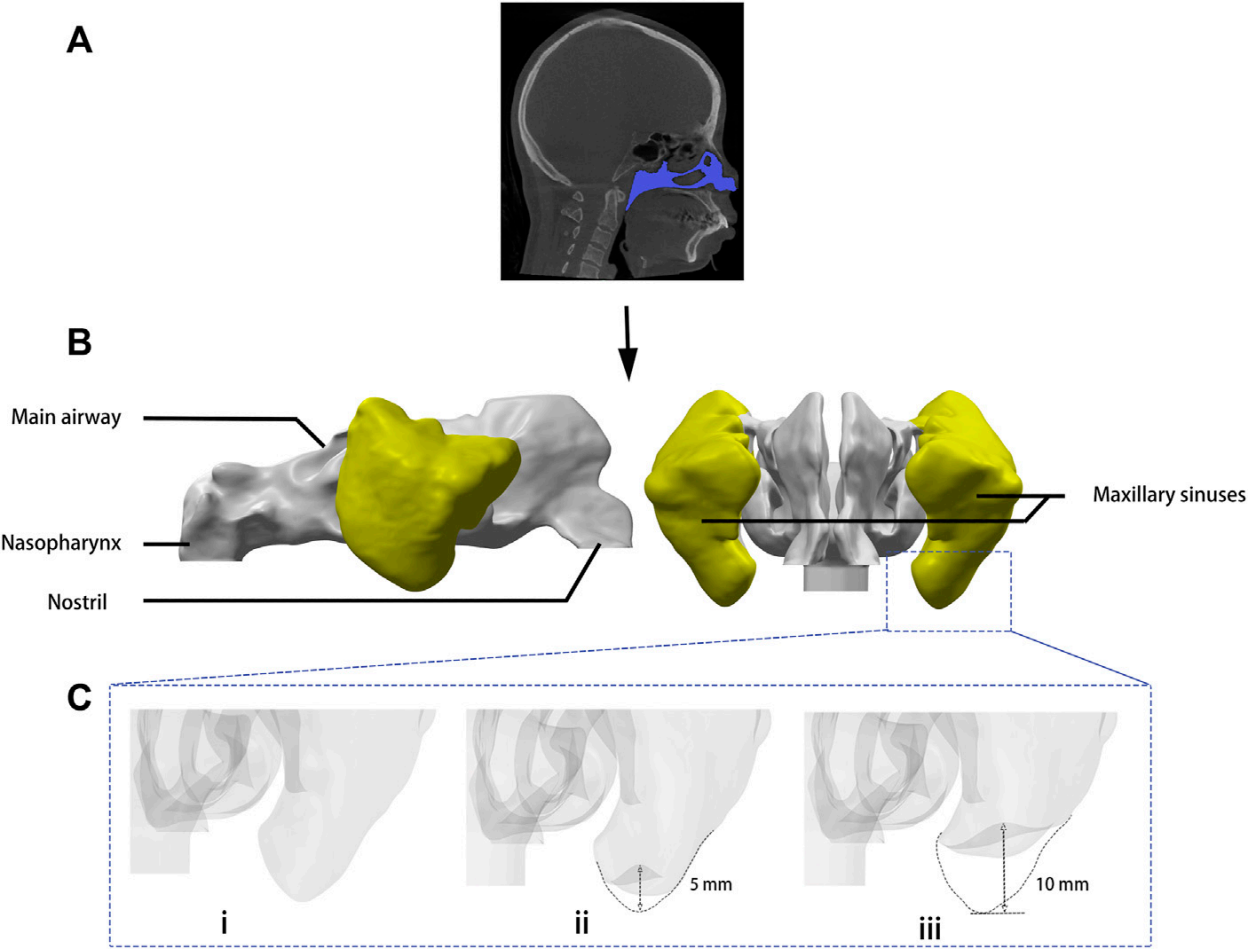
Digital modeling. **(A)** Images including the whole nasal cavity and maxillary sinuses were taken by cone beam computed tomography. **(B)** Three-dimensional model of the airspace was rebuilt by medical imaging software. **(C)** Sinus augmentation was simulated by computer-aided design software: (i) model A: sinus floor of the presurgical model, (ii) model B: sinus augmentation through the transcrestal approach, (iii) model C: sinus augmentation through the lateral window approach.

The morphological changes of the left maxillary sinus under different surgical techniques were simulated. As shown in Figure 1, the sinus floor of the pre-operative model (Model A) is ovoid in cross-section, and the transcrestal sinus elevation model (Model B) presents a mushroom at the center with the sinus floor lifted 5 mm, while the model of the sinus augmentation through the lateral window approach (Model C) was reconstructed into a wider range of convex, and the sinus floor was lifted 10 mm. The volumes of the left maxillary sinuses in model A, B, and C are respectively 11431.12, 10984.55, and 10491.9 mm^3^. The volumes of model B and C have been reduced by 3.90% and 8.21%, respectively, from the preoperative model (model A).

### Meshing

**FIGURE 2:**
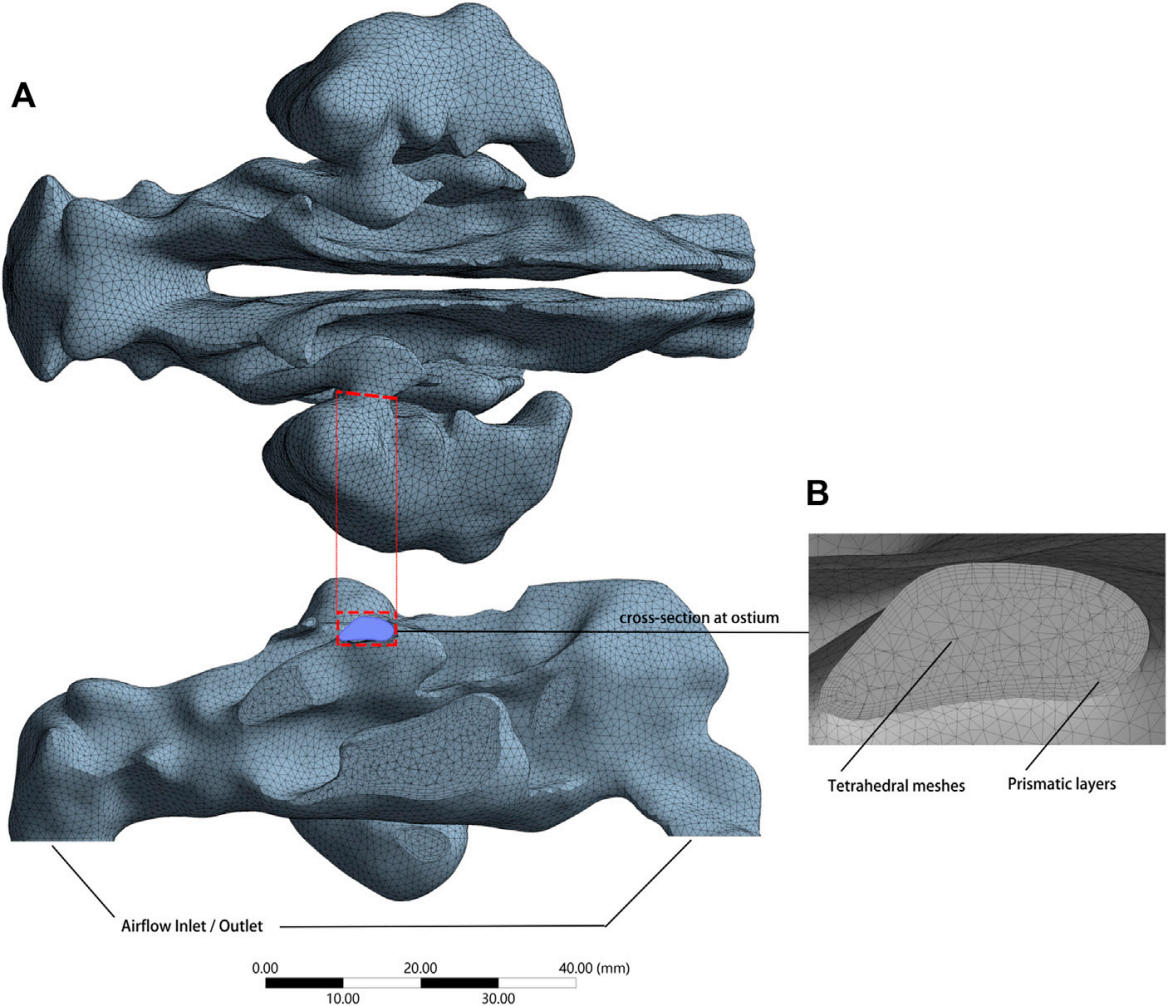
**(A)** Meshed model with an airflow inlet and outlet, consists of approximately 2,000,000 grids. **(B)** Cross-section plane at the ostium revealed tetrahedral elements constituted the main body and gradually thickening prism layers at the air space boundary.

Meshing procedure was carried out using ANSYS Meshing 2020 R2 (Swanson Analysis System Co., Houston, TX, United States). The main bodies of the models were meshed with tetrahedral elements. The narrower areas were improved with densified grids, and five gradually thickening prism layers were placed at the air space boundary (Figure 2).

### Boundary conditioning and numerical simulation

The planar surfaces around the nostril and the nasopharynx were respectively named as “outlet” and “inlet.” The boundary of the nasal cavity and the maxillary sinuses were defined as “wall.” CFD software package ANSYS Fluent 2020R2 was used to simulate the respiratory airflow inside the nasal cavity and maxillary sinuses, and the flow was assumed to be steady-state and turbulent. Navier-Stokes equation was numerically solved by k -ω model with a low Reynolds number correction and SIMPLEC algorithm (Garcia et al., 2010; Chenoweth et al., 2015; Chung et al., 2016). Table 1 summarized the boundary settings of the airflow rate. The steady expiratory flow rate of 250, 450, 788, and 1875 ml/s were respectively set up for the median expiratory flow rate, the peak expiratory flow rate of a breathing circle, the flow rate during sneezing, and the expiratory flow rate immediately after physical exercise. Inspiratory flow rate of 250, 450, and 720 ml/s were respectively set up for the median inspiratory flow rate, the peak inspiratory flow rate, and the flow rate during strong sniffing (Garcia et al., 2010; Chenoweth et al., 2015; Chung et al., 2016). The wall of the nasal cavity and the maxillary sinuses was assumed to be “rigid,” and “no-slip” condition was specified for the air-wall interface. The airflow temperature in the nasal airway and sinuses are thought to be constant between the room temperature and the body temperature in physiological state. Therefore, the effect of temperature variation along the airway was not considered in this simulation.

**TABLE 1:** Boundary airflow rates under different respiratory conditions.

Respiratory condition	Airflow rate
Median expiratory flow rate	Expiratory 250 ml/s
Peak expiratory flow rate	Expiratory 450 ml/s
Flow rate during sneezing	Expiratory 788 ml/s
Expiratory flow rate immediately after physical exercise	Expiratory 1875 ml/s
Median inspiratory flow rate	Inspiratory 250 ml/s
Peak inspiratory flow rate	Inspiratory 450 ml/s
Flow rate during strong sniffing	Inspiratory 720 ml/s

## Results

By digital modeling and CFD, the expiratory airflow within the nasal airspace and the maxillary sinuses was successfully simulated. The meshing procedure yielded ∼2 million tetrahedral elements for the pre- and post-operative models (Table 2). These quantities proved to be sufficient for numerically describing the airflow characteristics (Chen et al., 2011; Zang et al., 2012).

**TABLE 2:** Elements and nodes of meshed models.

	Element	Node
Model A	2286229	756161
Model B	2408273	807143
Model C	2434918	810624

### Airflow field within the maxillary sinuses

**FIGURE 3:**
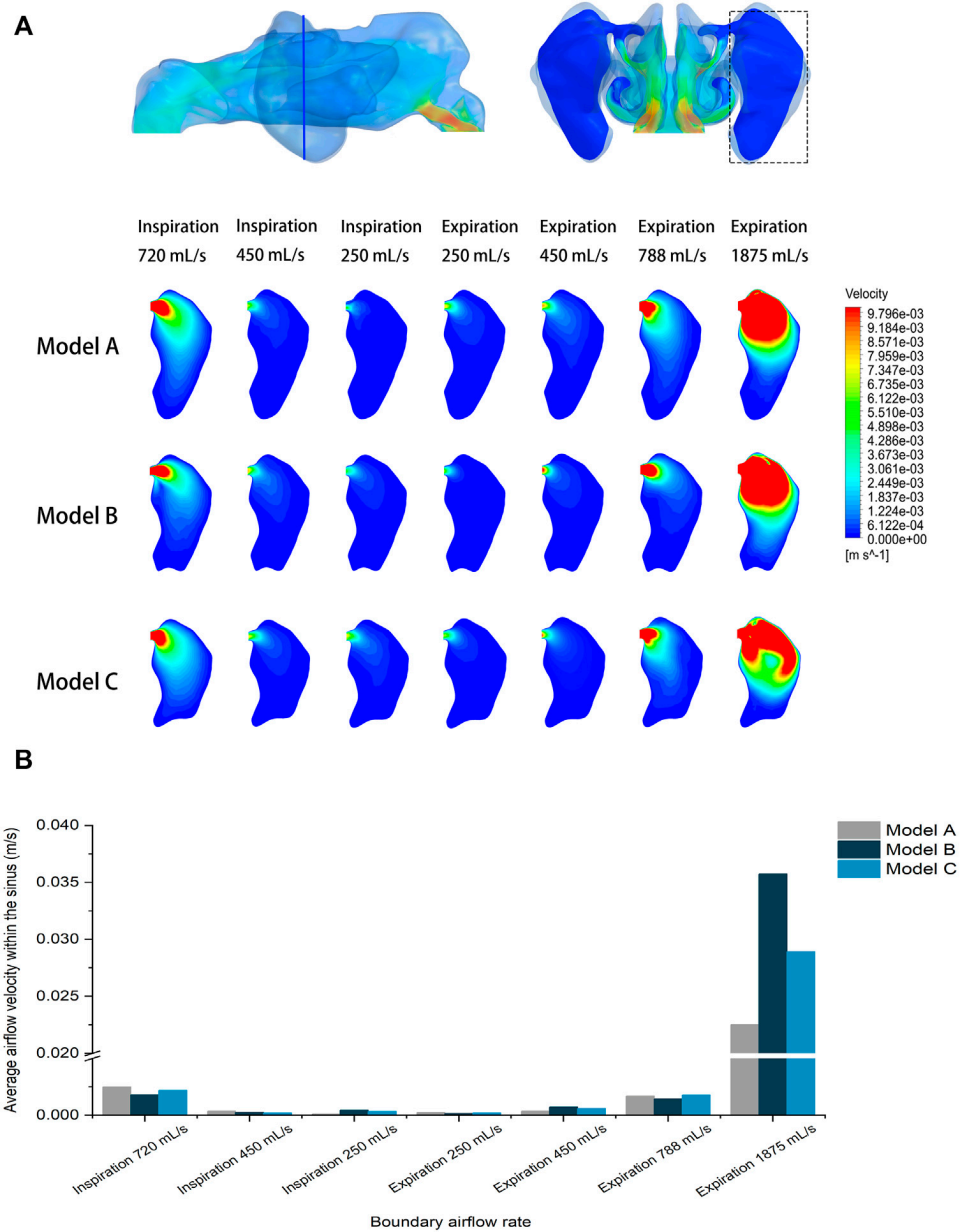
**(A)** Velocity distribution at the cross-section plane of the pre- and post-operative sinuses under different inlet flow rates. **(B)** Quantitative comparison of average airflow velocity within the pre- and post-operative sinuses under different flow rates.

**FIGURE 4:**
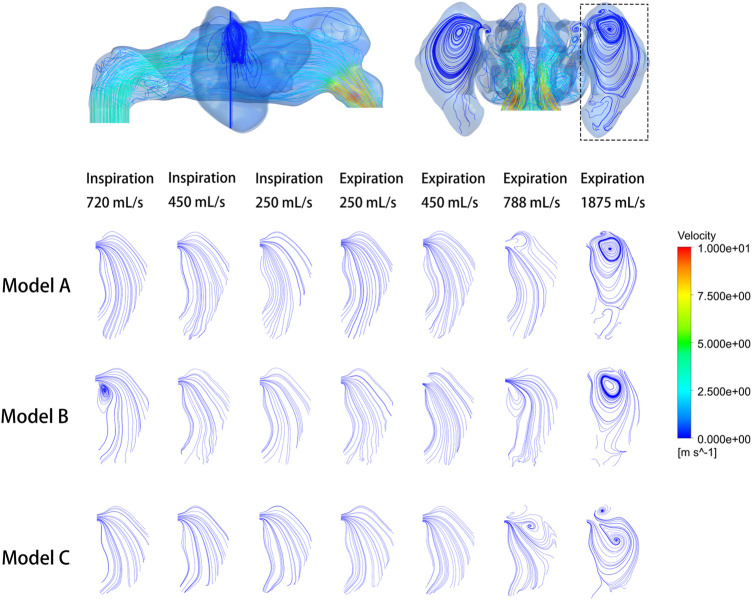
Velocity streamline at the cross-section plane of the pre- and post-operative sinuses under different inlet flow rates.

**FIGURE 5:**
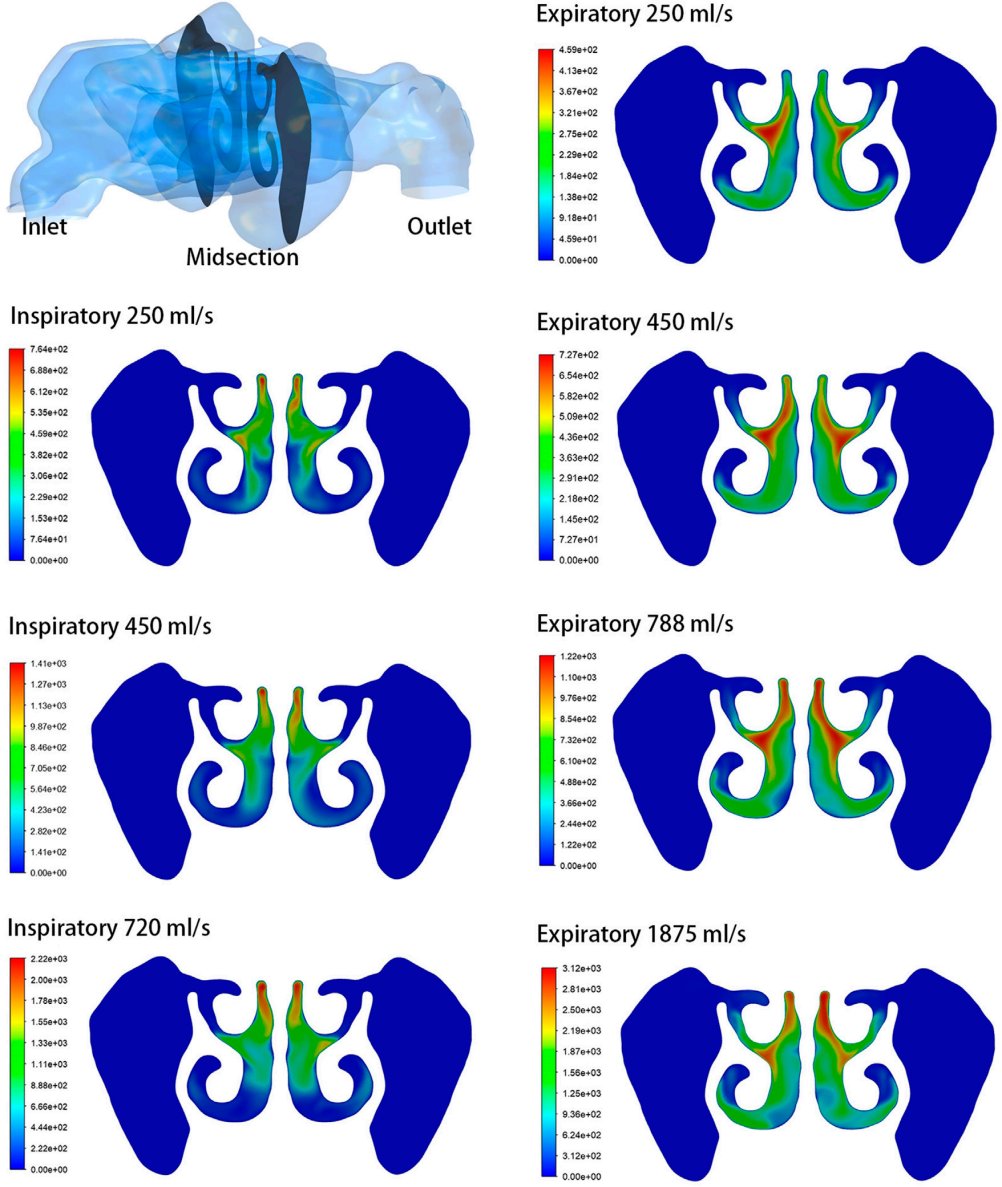
Contours of Reynolds number at the middle coronal section under different inlet flow rates.

The analysis focused on the airflow inside the left maxillary sinus where augmentation was performed. Compared to the boundary airflow velocity (1.9–14.25 m/s), the airflow velocity detected in the sinus dropped to an extremely low level. When the inlet flow rate was set at 1875 ml/s, the average velocity in the left maxillary sinus was 0.022–0.035 m/s, and the average airflow velocity within the sinus was generally below 0.002 m/s under other boundary settings, as shown in Figure 3. The pre- and post-operative model loaded by different expiratory flow rates demonstrated a similar airflow velocity distribution within the sinus. The main flow with a larger velocity was concentrated at the upper part, near the ostium, while a small amount of the airflow with minimum velocity reached the lower part of the sinus. The airflow streamline feature within the sinus was associated with the expiratory flow rate. For both the pre- and post-operative models, an annular airflow was detected in the sinus when the inlet flow rate was larger than 788 ml/s. When the flow rate was set at 250 ml/s or 450 ml/s during inspiration and expiration, the airflow streamline was laminar and diffused from the ostium to the sinus wall (Figure 4). The contours of Reynolds number at the middle coronal section under different inlet flow rates were calculated and visualized (Figure 5). Reynolds number was increased with the inlet flow rate. The largest RE (3120) was detected at the upper part of the nasal passage under an expiratory flow rate of 1875 ml/s. It is worth noting that RE stayed at a very low level within the sinuses under different respiratory conditions indicating that airflow in the maxillary sinus tends to be laminar.

### Wall pressure and wall shear stress

**FIGURE 6:**
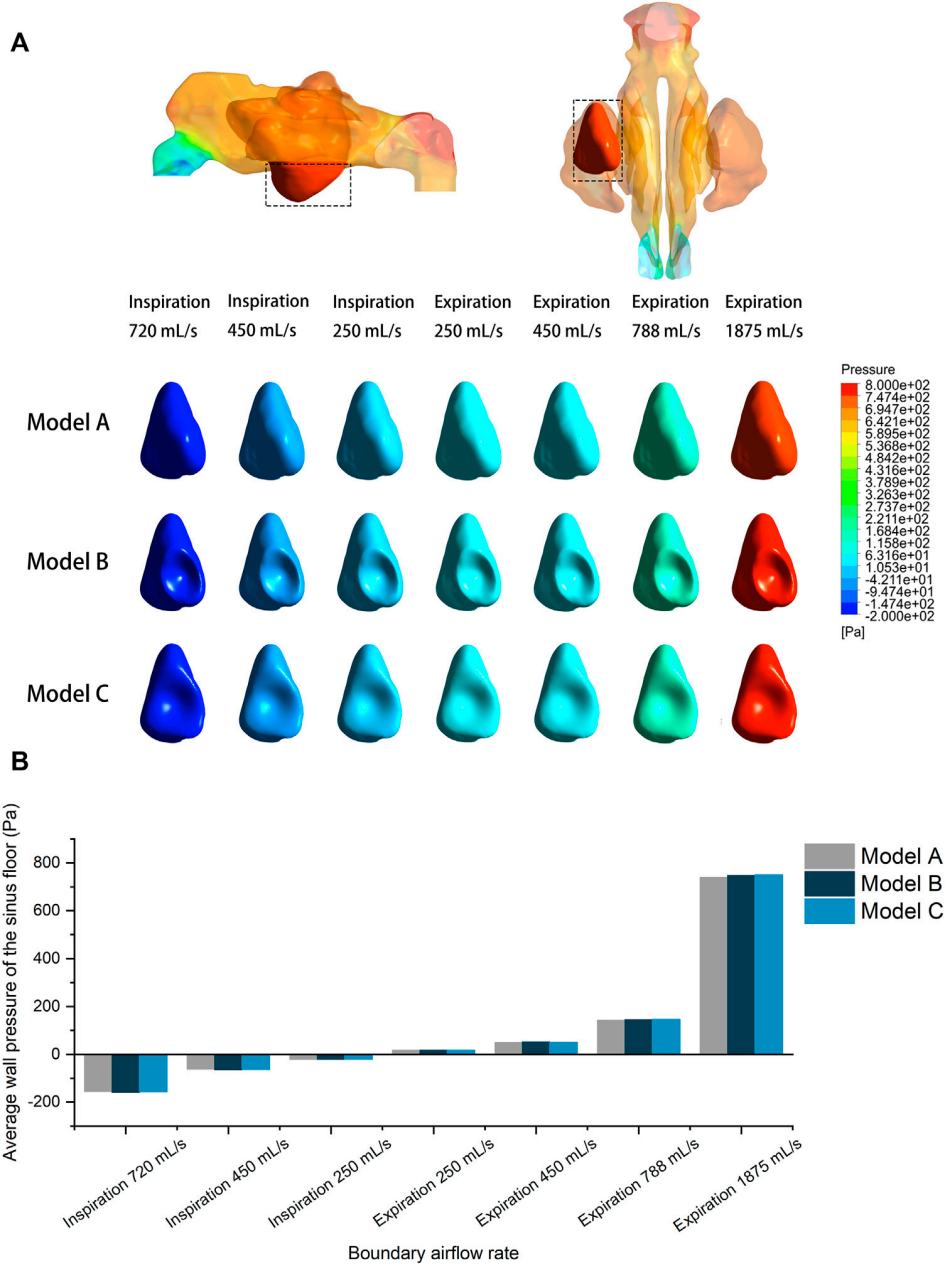
**(A)** Wall pressure contour of the pre- and post-operative sinus floor under different inlet flow rates. **(B)** Quantitative comparison of average wall pressure on the pre- and post-operative sinus floor under different flow rates.

Wall pressure of the airspace was simulated and visualized for both inspiratory and expiratory models. The sinus wall was respectively exerted with evenly distributed positive and negative pressure during expiration and inspiration. The surgical region of the left maxillary sinus floor was extracted for quantitative analysis. Figure 6 compares the pre- and post-operative wall pressure on the sinus floor at different inlet flow rates. While the absolute value of the average wall pressure increased with larger airflow rate, the morphological changes of the maxillary sinus following the augmentation barely influenced the wall pressure at the sinus floor during respiration. The difference between the wall pressures detected between the models is 3.05 Pa on an average, with a maximum difference of 9.51 Pa between Model A and Model C at an expiratory flow rate of 1875 ml/s. At the peak flow rate of calm inspiration and expiration (450 ml/s), the sinus floor was respectively under negative pressure of ∼62.65 Pa and positive pressure of ∼50.25 Pa. A negative pressure of ∼156 Pa was detected at the sinus floor in the simulation of sniffing. The largest positive pressure (∼745.29 Pa) was observed when the boundary flow rate was set at 1875 ml/s in the simulation of expiration immediately after physical exercise.

**FIGURE 7:**
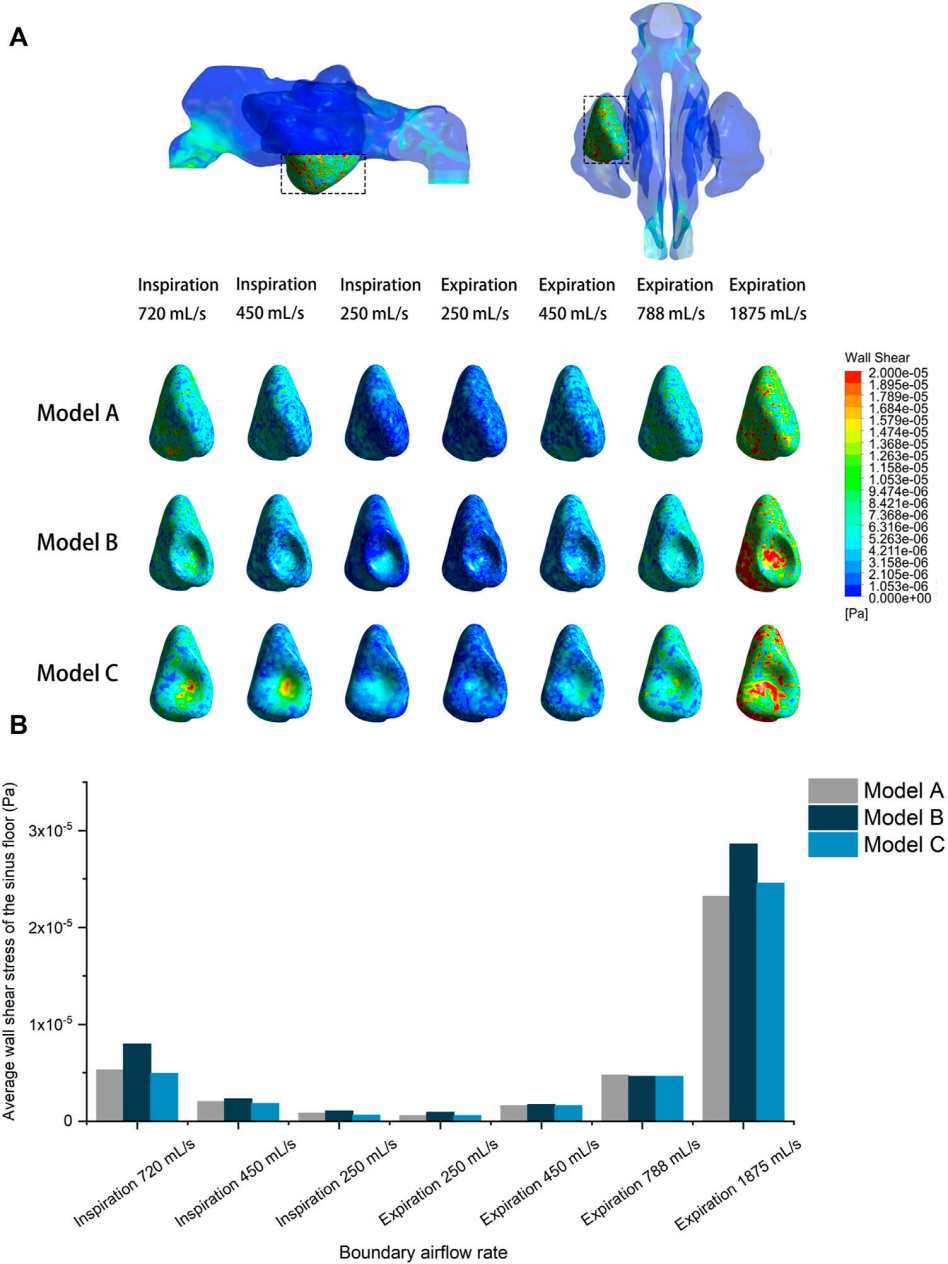
**(A)** Shear stress distribution of the pre- and post-operative sinus floor under different inlet flow rates. **(B)** Quantitative comparison of average wall shear stress on the pre- and post-operative sinus floor under different flow rates.

Figure 7 compares the pre- and post-operative average wall shear stress on the sinus floor at different inlet flow rates. It was observed that increased expiratory flow rate exerted greater shear stress on the sinus floor. However, all models recorded an extremely small magnitude of wall shear stress. The largest average value (2.86 × 10^–05^ Pa) was detected in Model B at a flow rate of 1875 ml/s. No significant difference was detected between the models, except that Model B recorded a slightly larger average wall shear stress than the other two models at the flow rate of 1875 ml/s.

Wall shear stress distributions on the left sinus floor were also visualized in different models. The wall shear stress on the sinus floor depicted a relatively uniform distribution in the pre-operative model, and the surgical elevation seems to have caused local concentration of the shear stress at the center where sinus floor was most lifted.

## Discussion

The purpose of maxillary sinus lift surgery is to increase the distance between the maxillary posterior sinus floor and the crest of the alveolar crest. Maxillary sinus lifting not only reduces the volume of the sinus but also changes the morphology at the bottom of the maxillary sinus. The present study attempted to evaluate the effect of mechanical stimulation on the sinus floor under different respiratory conditions and to investigate whether changes in the morphology of the sinus floor would alter airflow patterns and stress values.

CFD results showed that with or without surgical intervention, the portion of airflow entering the sinus cavity was negligible and the flow rate remained extremely low. Most of the airflow is circulated in the upper part of the sinus cavity. When the wall pressure loaded at the sinus floor increased with the upgraded inlet flow rate, the pre- and post-operative models demonstrated similar magnitudes of pressure at the sinus floor which is in agreement with Chung et al. (2014). They gave an explanation that the pressure gradient along the nasal passage was mainly associated with the gradient change in the flow area and was not directly affected by the ventilation of the maxillary sinus (Chung et al., 2014).

Previous studies have reported a significant reduction in the bone graft volume after maxillary sinus augmentation (Berberi et al., 2015; Gultekin et al., 2016), hence the purpose of this study was to investigate the role of mechanical stimulation in this specific setting. Up till now, research studies have used *in vitro* experiments to investigate the impact of hydrostatic pressure on human osteoprogenitor or osteoblast. For osteoblasts, short- or long-term cyclic hydrostatic pressure of 10–40 kPa at 0.3–1 Hz could elicit a positive osteogenic response (Roelofsen et al., 1995; Klein-Nulend et al., 1997; Nagatomi et al., 2003; Gardinier et al., 2009). For bone marrow-derived stem cells, short-term cyclic pressure of 10–36 kPa at 0.25 Hz, long-term cyclic pressure of 10 kPa at 2 Hz, or short-term hydrostatic pressure of 10–36 kPa is sufficient to stimulate osteogenic lineage commitment (Liu et al., 2009; Zhao et al., 2015; Sugimoto et al., 2017; Stavenschi et al., 2018). Studies have found that intermittent negative pressure of 50 kPa inhibits the proliferation of human mesenchymal stem cell, triggers cellular apoptosis, enhances osteogenesis activity, and induces the differentiation in osteoblast (Zhang et al., 2010; Yang et al., 2014). According to our simulated results, approximately 50 Pa negative pressure and positive pressure were detected at the sinus floor at the peak flow rate of respiration. The largest average wall pressure at the sinus floor was 748.9 Pa only at the most extreme expiratory condition (inlet flow rate of 1875 ml/s). These magnitudes are hundreds of times lower than the intramedullary pressure (4 kPa) induced by systemic blood pressure under static condition (Hillsley and Frangos, 1994). Therefore, the amount of pressure under different respiratory conditions is not adequate to stimulate or inhibit the maxillary sinus membrane stem cells to form bone.

In this study, we visualized and quantified the wall shear stress at the sinus floor. It was shown that the shear stress increased with the upgraded flow rate. The surgical interventions to the maxillary sinus seem to have caused local concentration of shear stress. However, the magnitude remained at an extremely low level. Interestingly, Bailie et al. (2009) found that elevated wall shear stress has a negative effect on nasal air conditioning, with inspiratory airflow leading to mucosal dryness and swelling (Bailie et al., 2009). Nonetheless, there is still no study to verify the magnitude with clinical significance of such mechanical irritation.

The limitations of this study include the following: The reductions in the sinus volume in post-operative models are relatively small (sinus in Model B: −3.90%, Model C: −8.21%), and that could be one of the factors leading to similar simulation results among different models. Moreover, using a single individual sinus to construct a model can be an issue since ventilation rate and airflow pattern can be influenced by different maxillary ostium size, cavity morphology, and volume. Therefore, the specific simulation results in this study using models from a single patient might not be universal to all populations and should be interpreted with caution. Nonetheless, the characteristics of airflow and wall pressure in maxillary sinus after sinus augmentation and the qualitative and quantitate results obtained from this study provides a better understanding of different respiratory conditions and anatomic change of the sinus cavity on fluid mechanical stimulation at the sinus floor. Future studies with larger samples and more individualized constructed models are needed to verify the current findings.

In summary, based on the fluid-dynamics calculations, the fluid-dynamic stimulation generated by respiratory airflow is minimal, and it can be concluded that the respiratory-induced pressure only has a limited influence on the sinus floor or elevated bone grafting.

## Data Availability

The original contributions presented in the study are included in the article/Supplementary Material; further inquiries can be directed to the corresponding authors.
