# Supplementary figures and images for: Characterizing the respiratory-induced mechanical stimulation at the maxillary sinus floor following sinus augmentation by computational fluid dynamics
Source: Front Bioeng Biotechnol. 2022 Jul 26;10:885130. doi: 10.3389/fbioe.2022.885130 (PMC9360545; doi:10.3389/fbioe.2022.885130)

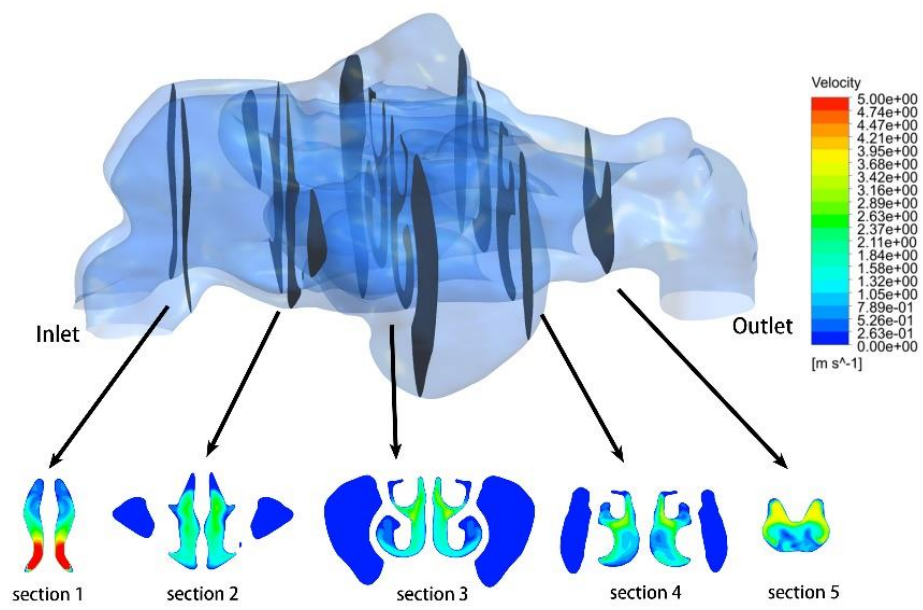

Figure S1. Cross sections of airflow velocity along the streamwise direction.

Supplement: Supplementary file 1 [file DataSheet1.PDF]
